# QSAR-derived affinity fingerprints (part 1): fingerprint construction and modeling performance for similarity searching, bioactivity classification and scaffold hopping

**DOI:** 10.1186/s13321-020-00443-6

**Published:** 2020-05-29

**Authors:** C. Škuta, I. Cortés-Ciriano, W. Dehaen, P. Kříž, G. J. P. van Westen, I. V. Tetko, A. Bender, D. Svozil

**Affiliations:** 1 CZ-OPENSCREEN: National Infrastructure for Chemical Biology, Institute of Molecular Genetics of the ASCR, v. v. i., Vídeňská 1083, 142 20 Prague 4, Czech Republic; 2 Centre for Molecular Informatics, Department of Chemistry, University of Cambridge, Lensfield Road, Cambridge, CB2 1EW UK; 3 CZ-OPENSCREEN: National Infrastructure for Chemical Biology, Department of Informatics and Chemistry, Faculty of Chemical Technology, University of Chemistry and Technology Prague, Technická 5, 166 28 Prague, Czech Republic; 4 Department of Mathematics, Faculty of Chemical Engineering, University of Chemistry and Technology Prague, Technická 5, 166 28 Prague, Czech Republic; 5 Computational Drug Discovery, Drug Discovery and Safety, LACDR, Leiden University, Einsteinweg 55, 2333 CC Leiden, The Netherlands; 6 Helmholtz Zentrum Muenchen – German Research Center for Environmental Health (GmbH) and BIGCHEM GmbH, Ingolstaedter Landstrasse 1, 85764 Neuherberg, Germany

**Keywords:** Affinity fingerprint, Biological fingerprint, QSAR, Similarity searching, Bioactivity modeling, Scaffold hopping

## Abstract

An affinity fingerprint is the vector consisting of compound’s affinity or potency against the reference panel of protein targets. Here, we present the QAFFP fingerprint, 440 elements long in silico QSAR-based affinity fingerprint, components of which are predicted by Random Forest regression models trained on bioactivity data from the ChEMBL database. Both real-valued (rv-QAFFP) and binary (b-QAFFP) versions of the QAFFP fingerprint were implemented and their performance in similarity searching, biological activity classification and scaffold hopping was assessed and compared to that of the 1024 bits long Morgan2 fingerprint (the RDKit implementation of the ECFP4 fingerprint). In both similarity searching and biological activity classification, the QAFFP fingerprint yields retrieval rates, measured by AUC (~ 0.65 and ~ 0.70 for similarity searching depending on data sets, and ~ 0.85 for classification) and EF5 (~ 4.67 and ~ 5.82 for similarity searching depending on data sets, and ~ 2.10 for classification), comparable to that of the Morgan2 fingerprint (similarity searching AUC of ~ 0.57 and ~ 0.66, and EF5 of ~ 4.09 and ~ 6.41, depending on data sets, classification AUC of ~ 0.87, and EF5 of ~ 2.16). However, the QAFFP fingerprint outperforms the Morgan2 fingerprint in scaffold hopping as it is able to retrieve 1146 out of existing 1749 scaffolds, while the Morgan2 fingerprint reveals only 864 scaffolds.
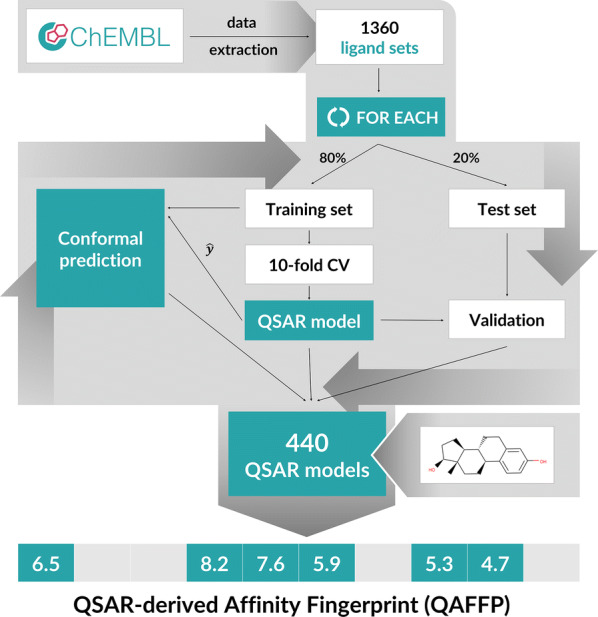

## Introduction

Virtual screening (VS) is a set of computational approaches used in the early stages of the drug discovery process. A major goal of VS is to reduce a chemical library to the manageable number of potentially active compounds [1]. In virtual screening, molecules are typically represented by molecular fingerprints [2], that reflect their chemical structure, or by chemical descriptors [3], that reflect their physico-chemical properties. However, the cellular response to a compound can be described without taking its chemical structure into account. Instead, the so-called bioactivity profile can be used to quantitatively describe compound interactions with the proteome [4, 5]. It was demonstrated that the comparison of compounds by their bioactivity profiles rather than by their structures can lead to the discovery of structurally dissimilar compounds eliciting same biological responses [6]. For example in the COMPARE approach [7, 8], GI50 data on 60 different human cancer cell lines were used to construct compound profiles and these enabled the discovery of structurally dissimilar compounds eliciting comparable bioactivities, often due to a shared mode of action [9, 10]. While the COMPARE profile is based on a cellular response, bioactivity profiles were also constructed using molecular target properties. In the so-called ‘affinity fingerprint approach’, 122 small molecules were encoded by their binding potencies against a reference panel of 8 proteins [11] and a regression model was used to predict compound potencies on two new targets. Analogously, “biospectra” consist of percentage inhibition values, measured at 10 µM concentration, across 92 ligand-binding GPCR, protease, ion channel and kinase assays [12]. Biospectra were successfully applied to predict agonism/antagonism of 24 dopamine-like compounds [12] and to investigate drug side-effects [13]. Apart from affinity fingerprints and biospectra, several other names for the description of a molecule using its experimentally determined bioactivity profile have been proposed: chemical genomic profile [14], chemical-genetic fingerprint [15] or activity spectrum [16, 17].

Although bioactivity profiling is a well-established methodology that was successfully applied for the discovery of several drug leads [18–20], its disadvantage is that dose-response data must be collected for all used targets. A cost saving alternative is to construct bioactivity profile using historically accumulated bioactivity data. In the first study of this kind [21], a diverse collection of 6000 small molecules showing potent antimalarial activity was identified by in silico compound profiling using bioactivity data from 131 unrelated cellular and enzymatic screens. In 2012, Petrone et al. [22] introduced the so-called HTS fingerprint (HTSFP) which is defined using bioactivity data from 195 biochemical and cellular assays historically run at Novartis. Petrone et al. [22] demonstrated that using the HTSFP fingerprint leads to the state-of-the-art performance in virtual screening and that the HTSFP fingerprint particularly excels in scaffold hopping. HTSFP’s potential was further demonstrated for mode-of-action analyses [23–26] and for the selection of activity and chemotype-enriched screening sets [24, 27, 28]. Though the HTSFP fingerprint enables compound comparisons on an unprecedented scale, it encounters one serious difficulty: a compound without the HTSFP fingerprint cannot be included in virtual screening. This problem is handled by Bioturbo similarity searching [24], in which a compound without a bioactivity profile is substituted by bioactivity profiles of structurally related compounds.

However, large screening collections, such as Novartis HTSFP data, are proprietary, which hampers academic laboratories and small companies to adopt affinity fingerprints in their computational workflows. To overcome these issues, recent studies used publicly available bioactivity data to classify biologically active compounds using affinity fingerprints. Riniker et al. [29] constructed a biological fingerprint using 95 assays publicly available in PubChem BioAssay repository [30]. When compared with ECFP4 fingerprints in classification tasks, this biological fingerprint led to better performance for the majority of assays. Similarly, the PubChem HTSFP [31] fingerprint consists of activities from 243 PubChem biochemical and cell-based assays spanning a large variety of target classes. Hit expansion experiments for 33 different targets yielded on average 27 times as many hits as a random selection with the average AUC of 0.82 and outperforming ECFP4 fingerprint for 29 targets [31].

The disadvantage of any experimentally-based affinity fingerprint is that a compound must be profiled across all fingerprint assays. A cost-efficient alternative is to evaluate compound activity in silico. For instance in DOCKSIM [32], affinity fingerprints were generated using DOCK [33] docking scores for the panel of 8 reference protein targets. This approach was later extended in the Flexsim-X method [34] by the application of flexible docking using the FlexX program [35] and by extending the panel of reference targets to 10. Other docking-based in silico profiling approaches include Drug Profile Matching [36–38] and Docking Score Index [39, 40].

The main disadvantages of docking-based approaches, namely the high computational footprint, the need for resolved protein structures and relatively low target space coverage, led to the development of the Bayes Affinity Fingerprint (BAF) [41]. In the BAF fingerprint, docking scores are replaced by Bayesian model scores, i.e. by probabilities that a ligand is active against a given set of targets. Models based on the BAF fingerprint improved retrieval rates in similarity searching across all activity classes on average by about 24% compared to the ECFP4 fingerprint [41].

For compound biological activity prediction, various Quantitative Structure-Activity Relationship (QSAR) methods have been developed [42–44]. Recently, several groups adopted QSAR models to predict compound activity across the human kinome and generated corresponding affinity fingerprints [45–48]. In the Profile-QSAR (pQSAR) method [45], Naïve Bayes models were trained on 115 Novartis proprietary kinase assays. Affinity fingerprints, constructed from Bayes activity probabilities, were than used to predict compound activity against kinases not included in the model yielding typically 20-fold to 40-fold enrichment of actives [45]. In pQSAR 2.0 [46], probabilities from Naïve Bayes models were replaced with IC50s predicted by Random Forest regression. Median correlation between predicted and experimentally measured IC50 increased from R^2^ = 0.24 in pQSAR 1.0 to R^2^ = 0.55 in pQSAR 2.0, making pQSAR 2.0 activity predictions comparable to medium throughput four-concentration IC50 measurements.

In addition to regression, binary QSAR (i.e., classification) was also utilized for the construction of affinity fingerprints. For example, binary affinity fingerprints were obtained using Random Forest classification models trained on the ligands of ~ 200 kinases [47]. Similarly, Balfer et al. [48] used Support Vector Machines to construct binary affinity fingerprints utilizing the panel of 24 different kinases implicated in diverse human cancers [49].

To capitalize on a large amount of bioactivity data available in the ChEMBL database [50, 51], we developed an in silico QSAR-based affinity fingerprint QAFFP. The QAFFP fingerprint was constructed using the predictions of high quality Random Forest models trained on freely available (i.e., non-proprietary) data covering diverse sets of molecular targets. Its performance was compared with that of the Morgan2 fingerprint (i.e., Morgan fingerprint with radius 2, the RDKit [52] implementation of the widely-used ECFP4 fingerprint [2]) for similarity searching, for the classification of compounds as active or inactive and for scaffold hopping. In addition to similarity searching, compound classification and scaffold hopping, QAFFP fingerprint was also applied in regression setting to predict compound in vitro potency, as described in the accompanying paper [REFERENCE GOES HERE].

## Methods

### Definitions

The biological activity of a compound is quantified either by its affinity (given as *Ki*/*Kd*), and/or by its potency (given as *IC50*/*EC50*). Affinity and potency measures are further referred to as activity types. In a given organism, one or more activity types can be measured for each distinct molecular target, defined by its unique Uniprot ID, and each organism/target/activity type combination is referred to as an assay. Throughout the manuscript, both potency and affinity values are included under the umbrella term “affinity fingerprint”.

### rv-QAFFP fingerprint construction

**Fig. 1:**
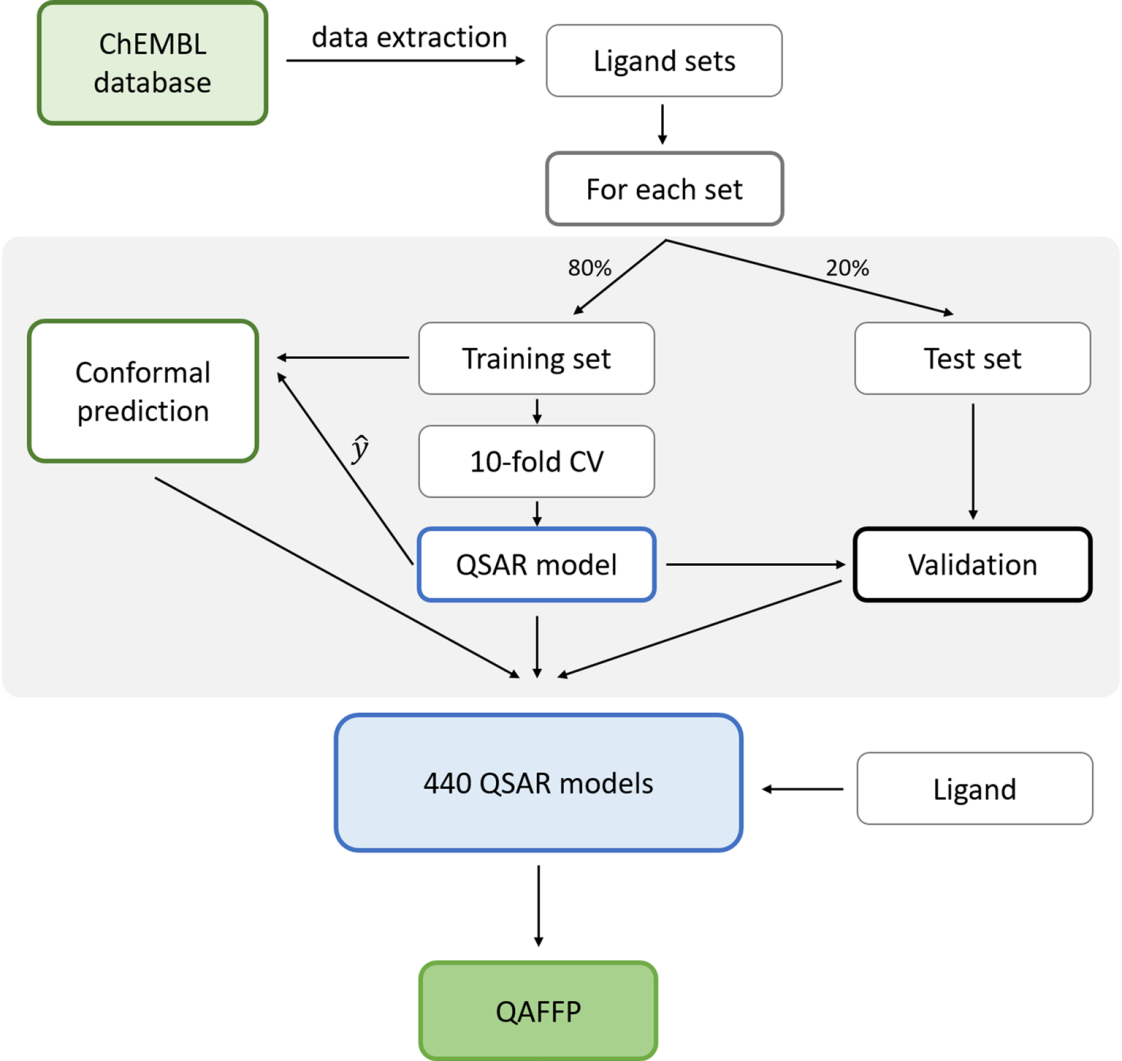
The workflow for the calculation of the rv-QAFFP fingerprint. 1360 ligand sets (Additional file 1) assayed against various molecular targets were extracted from the ChEMBL19 database [50, 51]. For each ligand set, Random Forest model was built using 80% of data for training and 20% for testing. Each QSAR model was validated using both internal (i.e., cross-validated) and external (i.e., test set) error measures and only models that satisfied stringent quality criteria were used for the construction of the rv-QAFFP fingerprint. The applicability domain of individual QSAR models was estimated using inductive conformal prediction [53–56]. The rv-QAFFP fingerprint is composed of 440 affinities predicted for the panel of assays covering 376 distinct molecular targets

The rv-QAFFP (rv stands for real valued) fingerprint of a compound is a vector containing compound’s biological activities across the panel of assays predicted by corresponding QSAR models. The workflow for the construction of the rv-QAFFP fingerprint is shown in Fig. 1 and described below.

QSAR models were built using publicly available data extracted from the ChEMBL database, version 19 [50, 51]. ChEMBL data are already extensively curated and standardized [57–59] using a pipeline [60] that includes salt stripping, neutralization and functional group normalization. QSAR models were obtained using both biochemical and cellular assay data, a strategy that proved to be successful in previous studies [22, 45]. To further increase the number of targets and the amount of training data, bioactivity data for both human and non-human targets were considered and separate models were built for individual organisms (Additional file 1). Only data sets satisfying the following criteria were considered: (i) activity type of *EC50, IC50, Ki* or *Kd*; (ii) activity relationship defined as “=”; (iii) ChEMBL confidence score equal to 7 or 9 (i.e., a ligand binds directly either to a subunit in a target complex or to a single protein). For QSAR modeling, only ligand sets with more than 50 distinct activity records were considered for further analysis. In the case where multiple activity values were annotated for the same ligand-target complex, their mean and standard deviation were calculated. The mean value was used as the activity value only if the standard deviation of all annotated measurements for a given compound-target system was lower than 0.5, otherwise the data point was discarded. A separate model was built for each assay resulting to the total number of 1360 models. Ligand sets combined for all 1360 models consist of 223,438 distinct compounds, with an average of 267 compounds per data set. The number of ligands used to train each QSAR model is given in Additional file 1.

To construct QSAR models, compounds were encoded using 1024 bits long Morgan2 fingerprint. For each of the 1360 ligand sets, a Random Forest (RF) regression model [61] was constructed using the module *ensemble.RandomForestRegressor* from the Python machine learning library scikit-learn [62]. The number of decision trees in the forest was set to 100 [53, 63, 64] and the maximum number of features to the total number of features. A higher number of trees (500) was also investigated, but no significant improvement was found (data not shown). Each data set was split into training and test sets in the 80:20 ratio using the stratified sampling of activity values. Each QSAR model was validated using the cross-validation correlation coefficient ${\text{q}}^{2}$, whereas the predictive power of the model on the test set (external validation) was evaluated using ${\text{R}}_{0 }^{ '2}$, the coefficient of determination for the predicted vs. the observed values constrained to pass through the origin:

$$q^{2} = 1 - \frac{{\mathop \sum \nolimits_{i = 1}^{N} \left( {y_{i} - \widehat{y}_{i} } \right)^{2} }}{{\mathop \sum \nolimits_{i = 1}^{N} \left( {y_{i} - \overline{y} } \right)^{2} }}$$

$${R^{\prime}}_{0}^{2} = 1 - \frac{{\mathop \sum \nolimits_{i = 1}^{N} \left( {y_{i} - \widehat{y}_{i}^{r0} } \right)^{2} }}{{\mathop \sum \nolimits_{i = 1}^{N} \left( {y_{i} - \overline{y} } \right)^{2} }}$$

where *N* is the size of the validation set (for $q^{2}$) or of the test set (for ${R^{\prime}}_{0 }^{2}$), $y_{i}$ are observed, $\widehat{y}_{i}$ predicted and $\overline{y}$ average activities, and $\widehat{y}_{i }^{r0} = k^{\prime}\widehat{y}$ where $k^{\prime} = \sum y_{i} \widehat{y}_{i} / \sum \widehat{y}_{i}^{2}$ is the slope of a predicted vs. observed regression line passing through an origin [65–67]. ${\text{q}}^{2}$ was estimated using tenfold cross validation of the training set. The training set was split into tenfolds of the same size using the stratified sampling of activity values. Because an RF algorithm incorporates random sampling, tenfold cross validation was repeated 10 times and the final ${\text{q}}^{2}$ was reported as the mean over all tenfolds in all 10 runs. The final model was constructed using the entire training set and its predictive power was assessed by calculating ${{\text{R}^{\prime}}}_{0 }^{2}$ for the test set.

**Fig. 2:**
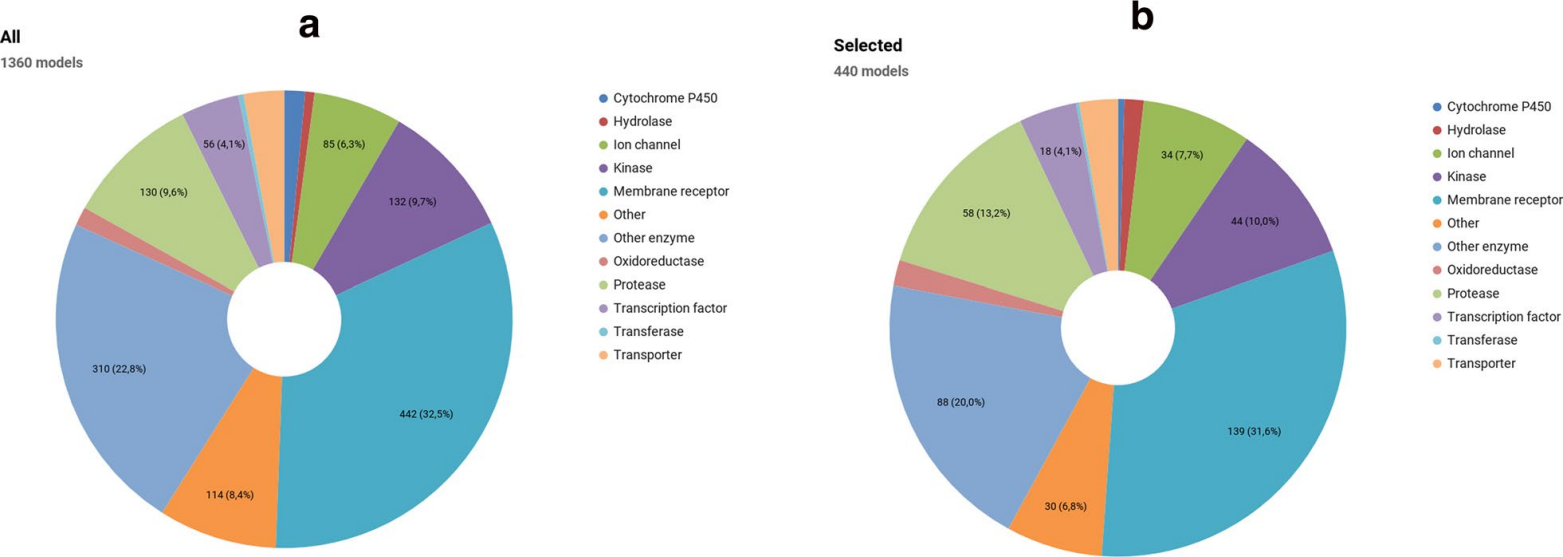
The representation of 12 target classes for all 1360 models and 440 models selected for the construction of QAFFP

Following previous recommendations for predictive bioactivity modeling [66, 68], only models with $q^{2} \ge 0.5$ and ${R^{\prime}}_{0}^{2} \ge 0.6$ were further considered for the construction of the QAFFP fingerprint. While the ${\text{q}}^{2}$ cut-off guarantees good fitting of the model to the training data, the ${{\text{R}^{\prime}}}_{0 }^{2}$ cut-off warrants a strong predictive power on new molecules (within the limits of a chemical diversity represented in a given data set). Although these thresholds may differ depending on modeling scenario [69] (e.g., higher errors can be tolerated in hit identification compared to lead optimization), they are, for the purpose of our study, stringent enough and provide a sufficiently high predictive power. Using these thresholds, 440 models, further referred to as *point prediction models*, out of the initial set of 1360 models were considered to be reliable and were used for the construction of the rv-QAFFP fingerprint (Additional file 1). The comparison of the representation of target classes between 1360 and 440 models (Fig. 2) shows that the assay space of 1360 models is evenly represented in 440 assays.

The QAFFP fingerprint of an unknown compound is obtained from the predictions of point prediction models applied on this compound. However, if the compound lies outside the Applicability Domain (AD) of the point prediction model, its biological activity might not be predicted reliably [70, 71]. Thus, it is important to estimate model AD. In the present work, the AD was estimated using the Conformal Prediction (CP) framework [53, 56].

A conformal predictor is the type of a confidence predictor that outputs, in contrast to a single value, a prediction interval with a guaranteed maximum error rate corresponding to a user-defined *confidence level*$1 - \varepsilon$, where $\varepsilon$ is called a *significance level*. For example, for a conformal regression model at 90% confidence level (i.e., at 10% significance level), at least 90% of all generated prediction intervals contain the correct value (i.e., no more than 10% of the actual values are outside the prediction interval). For each new compound, the *nonconformity score (measure)*$\alpha$ is calculated. The nonconformity score is the way of measuring how similar a new compound is to the training set compounds and it is defined as $\alpha = \frac{{\left| {y_{i} - \widehat{y}_{i} } \right|}}{{\lambda_{i} }}$ where $y_{i}$ is the observed bioactivity value, $\widehat{y}_{i}$ is the predicted bioactivity value and $\lambda_{i}$ is the scaling factor of the prediction interval. In the present work, a separate RF model, an *error prediction model*, was trained to predict the residual $\rho_{i}$ (i.e., the difference between the measured bioactivity and bioactivity predicted by the point prediction model), and this value was used as the scaling factor $\lambda_{i}$. The Conformal Predictor is then relating and ranking nonconformity scores of compounds to be predicted to scores of previously experimentally tested compounds. This is done by calculating a *p*-value (not to be confused with a *p*-value in statistical analysis), which is the fraction of existing compounds with nonconformity scores $\alpha$ smaller than is that of the new compound. If this fraction is small, the new compound is very non-conforming, *i.e.* rather different from previous compounds in the model, and it will hence have larger associated prediction ranges.

In this work, inductive conformal prediction (ICP) [72] was employed. In ICP, the training set is randomly divided into a “proper” training set and a “calibration” set. The model is trained using the proper training set and the calibration set is used to generate nonconformity scores $\alpha$. The disadvantage of ICP is that it requires more data because the calibration set instances must not be used to train the model. Therefore, we utilized cross-conformal prediction (CCP) [73] in which data are, similarly to cross-validation, divided in *k* folds (*k* equals 10 in the present work) and hence all training data are used as the training as well as the calibration set in turn.

### b-QAFFP fingerprint construction

A standard molecular representation used in similarity searching are binary fingerprints [74]. To compare the performance of rv-QAFFP with ECFP4 binary fingerprint [2], which has been established as a well-performing benchmark method in several previous studies [75–77], rv-QAFFP was converted to a binary form, b-QAFFP, using an activity cutoff and taking into account model AD. The predicted value was considered to lie within model AD if, at the given confidence level, the width of the prediction interval does not exceed a threshold the value of which was optimized. In b-QAFFP, all predicted values that lie above the activity cutoff and which are, at the same time, within model AD, were encoded as ones. All values that lie below the affinity cutoff but are still within model AD were encoded as zeros. Also, if the prediction lies outside model AD, the value was set to zero assuming that a compound is more likely to be inactive than active, similarly to what was reported in several previous studies [1, 29, 78].

### QAFFP performance assessment

The value of the QAFFP fingerprint was demonstrated for three common chemoinformatics applications: similarity searching, biological activity classification and scaffold hopping. In addition, the accompanying paper [REFERENCE GOES HERE] describes the application of QAFFP fingerprint in regression setting for the prediction of compound in vitro potency.

1. Similarity searching. In similarity searching, new potentially active compounds are identified by calculating their structural similarity [79, 80] to already known actives. This approach is based on a similar property relationship which states that structurally similar compounds possess similar properties [81]. Similarity searching is suitable if just one active compound is known. In similarity searching task, only the performance of the b-QAFFP fingerprint was evaluated.
2. Biological activity classification. In a biological activity classification, known actives and inactives are taken as an input to build a classification model that is used to classify unknown compounds. Typically, machine learning approaches are used as classifiers [82, 83]. Machine learning classification approaches are suitable if several actives are available. In a biological activity classification, the performance of both the rv-QAFFP and b-QAFFP fingerprints was evaluated.
3. Scaffold hopping. The aim of scaffold hopping is to discover active compounds that contain entirely new chemotypes [84–86]. The scaffold hopping potential was evaluated both for the rv-QAFFP and b-QAFFP fingerprints.

QAFFP fingerprint performance was assessed by two quality measures, *AUC* and *EF5*, the combination of which gives a good idea about the ability of the method to separate true positives from false positives [87]. *AUC* is the area under the ROC curve and it quantifies the general ability of a method to discriminate between actives and inactives [88]. *AUC* equals to the probability that a classifier will rank a randomly chosen positive instance higher than a randomly chosen negative example. However, *AUC* is not sufficiently sensitive to an early recognition [89], meaning that it does not prioritize models that place actives earlier in the ranked list of compounds. Thus, *AUC* was supplemented by the enrichment factor *EF* that explicitly measures the early recognition [89, 90]. *EF* is defined [91] as

$$EF\left( {\chi \% } \right) = \frac{{\frac{{P_{\chi \% } }}{{N_{\chi \% } }}}}{{\frac{{P_{total} }}{{N_{total} }}}}$$

where $\chi \%$ is the fraction of the sorted dataset *EF* is calculated for, $P_{\chi \% }$ is the number of actives in this fraction and $N_{\chi \% }$ is the number of all molecules in this fraction, $P_{total}$ is the number of actives in the data set and $N_{total}$ the number of all molecules in the data set. A method that is superior to a random selection of compounds returns *EF* > 1. In this study, *EF* at top 5% ($\chi$ = 0.05) of the sorted data set, abbreviated as *EF5*, was calculated.

QAFFP performance was compared to 1024 bits long Morgan2 fingerprint which is the equivalent of the ECFP4 fingerprint [2]. The ECFP4 fingerprint was chosen as a baseline for comparisons because of its high retrieval rates in various benchmarking studies [76, 92]. Differences in the performance between QAFFP and Morgan2 fingerprints were assessed by a one-sided exact Wilcoxon paired signed-rank test (a nonparametric alternative for the paired t-test) implemented in the R package coin [93, 94]. The effect size was assessed as a two-sided 95% confidence interval of the average difference of the criterion (*AUC* or *EF5*) between QAFFP and Morgan2 constructed by two-sided exact Wilcoxon signed-rank procedure.

### Similarity searching

The performance of the b-QAFFP fingerprint in similarity searching was assessed using the open-source benchmarking platform developed by Riniker et al. [90, 95]. The platform contains the lists of actives and inactives gathered from three different data collections (DUD [96], MUV [97] and ChEMBL [50, 51] subset proposed by Heikamp and Bajorath [98]), lists of predefined training sets, lists of randomly selected query molecules and the Python code needed to perform the evaluation. The availability of predefined training and test (i.e. query) molecules enables the easy reproduction of virtual screening experiments and the comparison of their results.

Two distinct data sets (Additional file 2) that simulate two following use cases are provided within the current version of the platform [95]:

1. Use case: a small set of diverse actives from a high-throughput screen is available. For this use case, heterogeneous data sets (further referred to as HET data sets, “data sets I” in the original publication [95]) consisting of 69 sets were assembled from the following three sources: 16 Maximum Unbiased Validation (MUV) data sets [97], 3 data sets from the Directory of Useful Decoys (DUD) [99], and 50 data sets extracted from ChEMBL [98].
2. Use case: a small set of related actives, i.e. compounds sharing one or two common scaffolds, from a publication or patent is available. For this use case, homogeneous data sets (further referred to as HOM data sets, “data sets II” in the original publication [95]) consisting of 37 sets were extracted from medicinal-chemistry papers that typically include data on one or two chemical series.

Both HET and HOM data sets contain assays that are also present in the QAFFP fingerprint and these were, thus, removed from the QAFFP fingerprint. Namely, out of 69 HET targets, 44 targets that correspond to 56 QAFFP assays are present in the QAFFP fingerprint (Additional file 2) which becomes, upon their removal, 384 bits long. Similarly, out of 37 HOM targets, 27 targets that correspond to 38 QAFFP assays are present in the QAFFP fingerprint (Additional file 2) which becomes, upon their removal, 402 bits long.

For each target of three data-set collections (MUV, DUD, ChEMBL), two compound lists are provided within the benchmarking platform, one for actives and one for inactives. For HET data sets, the VS experiment was repeated 50 times for each target, with different randomly selected training sets. To ensure the reproducibility of the results, the precalculated training sets are provided as the part of the benchmarking platform. In our case, each training set consisted of 10 actives (further referred to as query molecules) and of 20% of randomly selected inactives. The remaining actives and inactives formed the test set. For each molecule in the test set, its similarity to query molecules was calculated and only the highest similarity value was considered, corresponding to the MAX fusion rule [100]. The whole test set was then sorted by the decreasing similarity and *AUC* and *EF5* were calculated from this ranked list.

For HOM data sets, the VS experiment was performed once for each paper using, as the training set, all actives from the paper and 10% of the inactives. The test set consisted of 99 actives from the benchmarking data set for the same target and the rest of the inactives.

The similarity between molecules was evaluated by the Rogot-Goldberg index *s*_RG_ [101]

$$s_{\text{RG}} = \frac{a}{2a + b + c} + \frac{d}{2d + b + c}$$

where *a* is the number of bits set to 1 in both objects, *d* is the number of bits set to 0 in both objects, *b* is the number of cases in which bits in the first objects are set to 1 and bits in the second object are, at the same time, set to 0, and *c* is the number of events in which bits in the first objects are set to 0 and bits in the second object are, at the same time, set to 1. The Rogot-Goldberg index represents an efficient alternative [102] to the commonly used Tanimoto index, however, it takes into account not only bits set to 1, but also bits set to 0. For the b-QAFFP fingerprint, the Rogot-Goldberg index is more realistic measure than the Tanimoto index because the information at which targets the compound is active is equally important as the information at which targets it is inactive.

To compare the performance of the b-QAFFP and Morgan2 fingerprints, both types of fingerprints were calculated for HET and HOM data sets. The affinity cutoffs of 5 (i.e., 10 µM), 6 (i.e., 1 µM), 7 (i.e., 100 nM) and 8 (i.e., 10 nM) were used for the construction of the b-QAFFP fingerprint. Model AD was estimated using conformal prediction, but the case of not using AD was also considered. An ICP was used with the confidence level of 90% and the maximum interval width, that distinguishes whether the prediction is reliable enough, was set to 4.0 (i.e., predicted value ± 2.0). For every data set and every type of fingerprint, a separate model was trained and its performance was assessed by calculating the *AUC* and *EF5* values for the test set.

### Biological activity classification

Both HET and HOM sets are highly imbalanced with a considerably higher amount of inactives (e.g., MUV data sets contain 30 actives and 15,000 inactives, see Additional file 2), which limits their utility for the training of a classification model. Thus, new data sets, further referred to as the CLASS data sets, were constructed from 920 assays that were not used for QAFFP construction. The CLASS data sets were selected using the following criteria: (1) compounds with a potency ≤ 5 were considered as inactive, compounds with a potency ≥ 6 as active, (2) for every CLASS data set (assay), more than 60 inactives and more than 60 actives must be available (3) only assays that share no more than 10% of ligands with any of QAFFP assays were included in the CLASS data sets. The CLASS data sets consist of 23 assays (21 *IC50* and 2 *EC50*) covering 23 targets (Additional file 3).

To construct the rv-QAFFP fingerprint, the AD was estimated by an ICP. If the prediction interval width for the given data point was larger than ± 2.0 at the confidence level of 90%, the prediction was considered unreliable and it was replaced by an average of all reliably predicted affinities. The b-QAFFP fingerprint was constructed using several affinity cutoffs (5 (i.e., 10 µM), 6 (i.e., 1 µM), 7 (i.e., 100 nM) and 8 (i.e., 10 nM)) considering or not considering the AD that was estimated by an ICP using the confidence level of 90% and the maximum interval width was set to 4.0 (i.e., predicted value ± 2.0). All compounds lying outside the AD were substituted with zeros.

In the biological activity classification task, the CLASS data sets were used to train RF models to classify ligands as active or inactive. Because some CLASS data sets are imbalanced (Additional file 3), a balanced RF model [103] from the imbalanced-learn Python package [79] was trained. Each RF model consisted of 100 trees [53, 63, 64] and GINI index was used as a purity criterion to split a node. Ligands were encoded by the Morgan2 (1024 bits long), rv-QAFFP and b-QAFFP (both 440 bits long) fingerprints. fivefold cross-validation was used to evaluate model performance. Each cross-validation was repeated 10-times and final results were averaged over all repetitions and all splits.

### Scaffold hopping

Scaffold hopping was benchmarked using the CLASS data sets (Additional file 4) with one set removed (ChEMBL ID: CHEMBL5313) as it did not contain enough scaffolds. Ligands were encoded by the Morgan2 (1024 bits long), rv-QAFFP and b-QAFFP (both 440 bits long) fingerprints. The following settings were used to construct the rv-QAFFP and b-QAFFP fingerprints:

1. rv-QAFFP—RF models were trained on raw data considering or not considering model AD estimated by an ICP. At the confidence level of 90%, if the prediction interval width was larger than ± 2.0, the prediction was considered unreliable and it was replaced by an average of all reliably predicted affinities.
2. b-QAFFP—the affinity cutoffs of 5 (i.e., 10 µM), 6 (i.e., 1 µM), 7 (i.e., 100 nM) and 8 (i.e., 10 nM) were used for the construction of the b-QAFFP fingerprint. Model AD was estimated by ICP, but the case of not using AD was also considered. An ICP was used with the confidence level of 90% and with the maximum interval width of ± 2.0. Ligands lying outside model AD were considered to be inactive (i.e., corresponding bits were set to 0).

For each ligand, its cyclic skeleton (CSK) [104] was generated. The CSK, also known as a graph framework [105], is derived from the Bemis and Murcko (BM) scaffold [105] by converting all heteroatoms to carbon and setting all bond orders to 1. Compound was considered to be active if the negative decadic logarithm of its potency was higher than 6. The CSKs of all active compounds in a given assay are further referred to as active CSKs (ACSKs). In addition, active CSKs with at least five active compounds are further referred to as rich active CSKs (RACSKs). For the given assay, the training set was formed by compounds from one RACSK plus all inactive compounds. The test set then consisted of all remaining active compounds. Thus, the number of training sets for each assay equals to the number of its RACSKs (Additional file 4). Using each training set, a balanced RF model [79, 103] was constructed and applied on the test set. Compounds in the test set were classified either as active or as inactive using the probability threshold of 0.5. For each classified active, its CSK was retrieved and the number of unique CSKs, summed over all training data sets, was calculated for each assay. The scaffold hopping potential was assessed for the Morgan2, rv-QAFFP and b-QAFFP fingerprints. In addition, ACSKs retrieved using both rv- and b-QAFFP fingerprints were pooled and reported as rv+b-QAFFP.

## Results and discussion

### Data statistics

440 QSAR models used for the QAFFP construction were built using 256 *IC50,* 137 *Ki, 37 EC50 and 10 Kd* assays that cover 376 distinct molecular targets; i.e. 64 targets were modeled with more than one assay. However, these “duplicates” are not redundant as the maximum Pearson correlation coefficient between two assays belonging to the same target was only 0.53. 376 targets originate from 34 organisms (Additional file 1), a majority comes from human (254 targets) followed by rat (45 targets) and mouse (18 targets).

### Performance of b-QAFFP fingerprint in similarity searching

The results of the evaluation of various approaches for the construction of the b-QAFFP fingerprint are given in the Table 1 for HET data sets, and in the Table 2 for HOM data sets. Further details can be found in the Additional files 5 and 6, Figs. 1 and 2.

**Table 1:** The comparison of the performance of the Morgan2 (ECFP4) and b-QAFFP fingerprints for similarity searching for 69 HET data sets

FP	Morgan2	b-QAFFP							
AD	–	No				Yes			
Cutoff	–	5	6	7	8	*5*	6	7	8
AUC	0.66 ± 0.01	0.63 ± 0.01	0.63 ± 0.01	0.65 ± 0.01	0.58 ± 0.01	*0.70* ± *0.01*	0.62 ± 0.01	0.63 ± 0.01	0.56 ± 0.01
EF5	6.41 ± 0.40	3.67 ± 0.25	4.52 ± 0.33	4.50 ± 0.30	2.27 ± 0.16	*5.82* ± *0.34*	4.65 ± 0.32	3.97 ± 0.26	1.76 ± 0.12

Model AD was estimated by an ICP. Affinities predicted to lie outside model AD were encoded by zeros. Various affinity cutoffs were used to construct the b-QAFFP fingerprint. Best results are shown in a column in italic. Data shown are averages over all HET data sets with their standard errors of the mean. The b-QAFFP fingerprint is 384 bits long

**Table 2:** The comparison of the performance of the Morgan2 (ECFP4) and b-QAFFP fingerprints for similarity searching for 37 HOM data sets

FP	Morgan2	b-QAFFP							
AD	–	No				Yes			
Cutoff	–	5	6	7	8	*5*	6	7	8
AUC	0.57 ± 0.02	0.61 ± 0.02	0.58 ± 0.03	0.61 ± 0.02	0.57 ± 0.02	*0.65* ± *0.02*	0.59 ± 0.02	0.61 ± 0.02	0.56 ± 0.02
EF5	4.09 ± 0.42	3.44 ± 0.30	3.52 ± 0.47	3.88 ± 0.54	2.33 ± 0.24	*4.67* ± *0.53*	3.56 ± 0.51	3.39 ± 0.53	1.81 ± 0.21

Model AD was estimated by an ICP. Affinities predicted to lie outside model AD were encoded by zeros. Various affinity cutoffs were used to construct the b-QAFFP fingerprint. Best results are shown in a column in italic. Data shown are averages over all HOM data sets with their standard errors of the mean. The b-QAFFP fingerprint is 402 bits long

The Tables 1 and 2 show that the best setting for the construction of the b-QFFP fingerprint is to estimate the AD with an ICP and to use the affinity cutoff of 5 (i.e., 10 µM). While at this setting the b-QAFFP fingerprint yields statistically significantly better *AUC* both for the HET and HOM data sets (verified by the one-sided exact Wilcoxon signed-rank test with the p-value for alternative hypothesis Morgan2 < QAFFP being p-value = 7.50e−04 for HET and p-value = 5.79e−07 for HOM), *EF5* is significantly better for the Morgan2 fingerprint in the case of the HET data sets (p-value is 6.70e−04 for alternative Morgan2 > QAFFP) and there are no significant differences in *EF5* between the b-QAFFP and Morgan2 fingerprints for the HOM data sets (p-value = 0.21 for two-sided alternative Morgan2 ≠ QAFFP). The corresponding nonparametric 95% confidence intervals reveal that the average excess of b-QAFFP’s *AUC* over Morgan2’s *AUC* can be expected in the range from 0.12 to 0.49 for the HET data sets and from 0.58 to 0.12 for the HOM data sets. On the other hand, the 95% nonparametric confidence interval for the average excess of Morgan2’s *EF5* over b-QAFFP’s *EF5* shows the effect size ranging from 0.28 to 0.69 for the HET sets. The analysis of nonparametric 95% confidence intervals for differences between two fingerprints shows, that though b-QAFFP yields significantly better values than Morgan2 for some measures and vice versa, the effect size is relatively small. Thus, it may be concluded that the b-QAFFP and Morgan2 fingerprints they provide comparable results in similarity searching.

### Performance of b-QAFFP and rv-QAFFP fingerprints in biological activity classification

**Fig. 3:**
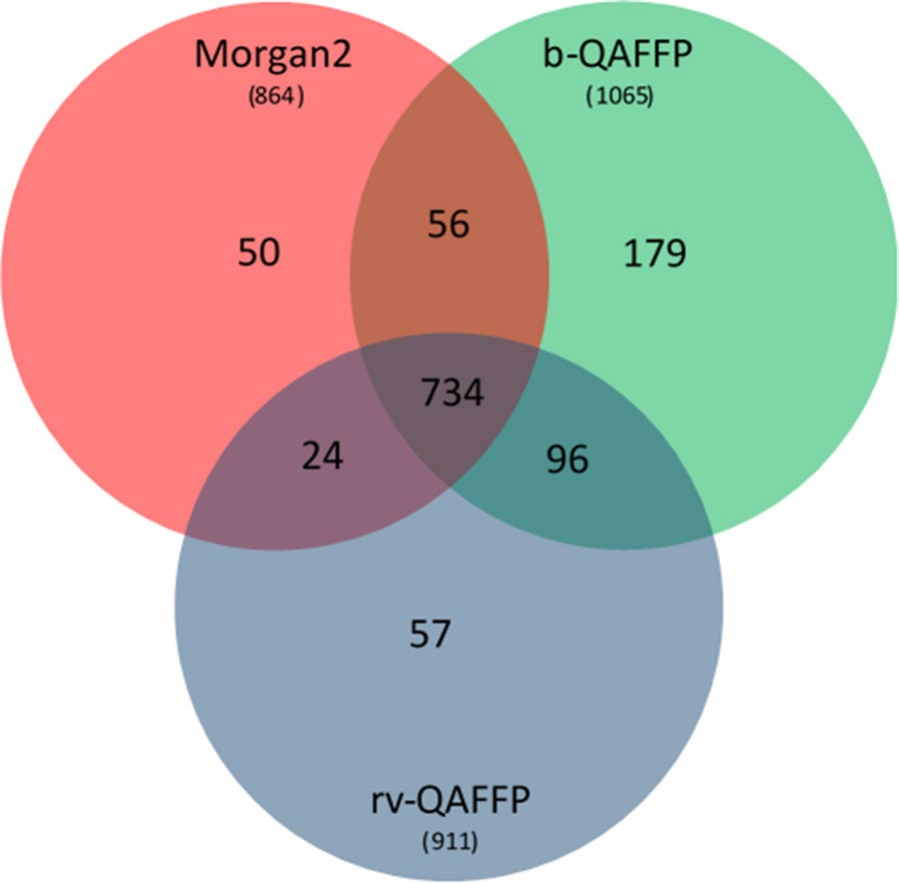
The number of ACSKs identified by the Morgan2, b-QAFFP and rv-QAFFP fingerprints. The total number of ACSKs in the CLASS data sets is 1749

**Fig. 4:**
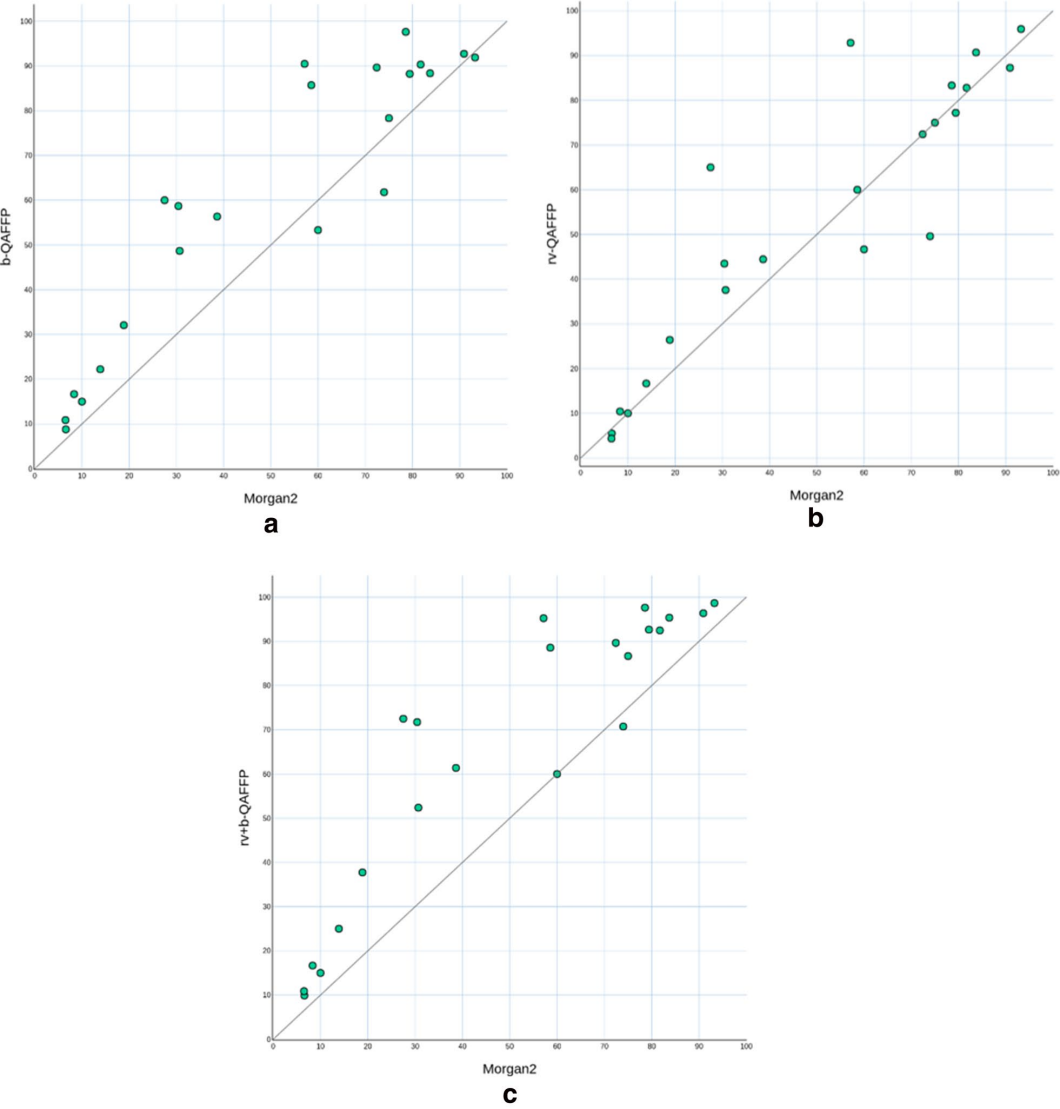
ACSKs recall using the b-QAFFP (**a**) and rv-QAFFP fingerprints (**b**) and their combination rv+b-QAFFP (**c**). Recall is the percentage of ACSKs revealed from all ACSKs existing in the given data set

For every of the 23 CLASS data sets and for every type of fingerprint, a separate RF model was trained and its performance was assessed by calculating the *AUC* and *EF5* for the test set. In addition, rv-QAFFP models were trained using both raw and Z-standardized data (i.e., all data points were converted to their Z-values), but no significant differences between these two approaches were identified. Average value for each quality measure, together with its standard error of the mean, is given in the Table 3. Further details can be found in the Additional files 6 and 7, Figs. 3 and 4.

**Table 3:** The comparison of the performance of the Morgan2 (ECFP4), rv-QAFFP and b-QAFFP fingerprints for biological activity classification of 23 CLASS data sets

FP	Morgan2	rv-QAFFP		b-QAFFP							
AD	–	No	Yes	No				Yes			
Cutoff	–	–	–	5	6	7	8	*5*	6	7	8
AUC	0.87 ± 0.01	*0.86* ± *0.01*	0.86 ± 0.02	0.83 ± 0.01	0.85 ± 0.01	0.84 ± 0.02	0.77 ± 0.01	*0.85* ± *0.02*	0.85 ± 0.02	0.83 ± 0.02	0.73 ± 0.01
EF5	2.16 ± 0.16	*2.08* ± *0.14*	2.08 ± 0.13	2.03 ± 0.13	2.09 ± 0.14	2.08 ± 0.14	1.89 ± 0.11	*2.10* ± *0.14*	2.08 ± 0.14	2.04 ± 0.13	1.78 ± 0.10

Model AD was estimated by an ICP with the confidence level of 90%. rv-QAFFP models were trained using raw data. Considering AD for rv-QAFFP means that if the prediction interval width was larger than ± 2.0, the prediction was regarded unreliable and was replaced by the average of all reliably predicted affinities. Various affinity cutoffs were used to construct the b-QAFFP fingerprint. Affinities predicted to lie outside model AD were encoded by zeros. Best results are shown in columns in italic. Data shown are averages over all CLASS data sets with their standard errors of the mean. Both rv-QAFFP and b-QAFFP fingerprints are 440 bits long

The highest rv-QAFFP’s *AUC* is achieved if the rv-QAFFP fingerprint is constructed from models trained on raw data without regard to their ADs. The difference between considering and not considering the AD lies in a way data points outside the AD are treated. When the AD is taken into account, these data points are imputed by the mean over all reliably predicted affinities. If the AD is not considered, these data points are filled in by predicted affinities, though estimated with less confidence.

The recommended settings for b-QAFFP fingerprint construction are same as those identified for similarity searching, i.e. to estimate the AD with an ICP and to use the affinity cutoff of 5 (i.e., 10 µM). At these settings, both b-QAFFP and rv-QAFFP fingerprints perform, in terms of *AUC*, significantly worse than the Morgan2 fingerprint (p-value of the signed-ranked paired test for alternative Morgan2 > QAFFP is p-value = 3.58e−07 for both b-QAFFP and rv-QAFFP). The average deficit of b-QAFFP *AUC* compared to Morgan2 *AUC* can be expected in the range 0.01–0.03 (with 95% confidence) and the average deficit of rv-QAFFP *AUC* to Morgan2 *AUC* in the range 0.01–0.02 (with 95% confidence). However, these differences can be considered as small, compared to the average *AUC* value of ~ 0.86. In terms of *EF5*, no statistically significant differences were detected between the Morgan2, b-QAFFP and rv-QAFFP fingerprints. Therefore, the performance of the QAFFP fingerprints can be considered comparable to that of the Morgan2 fingerprint also for biological activity classification.

### Performance of b-QAFFP and rv-QAFFP fingerprints in scaffold hopping

For every of the 22 CLASS data sets (one CLASS data set was not used as it contained no RACSK) and for every type of fingerprint, a separate RF model was trained and its performance was assessed by calculating the average number of ACSKs per an assay (Table 4).

**Table 4:** The average number of ACSKs per an assay (and its standard error of the mean SEM) in 22 CLASS sets revealed by the Morgan2, rv-QAFFP and b-QAFFP fingerprints

FP	Morgan2	rv-QAFFP		b-QAFFP							
AD		No	Yes	No				Yes			
Cutoff	–	–	–	5	6	7	8	*5*	6	7	8
Average	39.27	*41.41*	41.45	48.40	47.80	48.89	66.14	*48.41*	47.89	48.54	67.58
SEM	8.25	*8.51*	8.68	10.26	10.47	11.18	16.02	*10.37*	10.54	10.88	16.84

Model AD was estimated by an ICP with the confidence level of 90%. rv-QAFFP models were trained using raw data. Considering AD for rv-QAFFP means that if the prediction interval width was larger than ± 2.0, the prediction was regarded unreliable and was replaced by the average of all reliably predicted affinities. Various affinity cutoffs were used to construct the b-QAFFP fingerprint. Affinities predicted to lie outside model AD were encoded by zeros. Data shown are averages over 22 CLASS data sets with their standard errors of the mean (SEM). Both rv-QAFFP and b-QAFFP fingerprints are 440 bits long. The recommended settings are shown in columns in italic

Table 4 reveals that for both rv-QAFFP and b-QAFFP there are no differences in the performance whether AD is considered or not. The average number of recovered ACSKs is lower for rv-QAFFP compared to b-QFFP. For b-QAFFP, the results are pretty stable for thresholds of 5, 6 and 7. Only for threshold 8, a significant increase in the average number of recovered ACSKs can be observed. Threshold 8 means that only very potent molecules (< 10 nM) are considered as active, at this threshold b-QAFFP bit density dramatically drops (Table 5) and data become too sparse. For sparse data, it’s very likely that for some RF node the bootstrapped sample and the random subset of features will play along to produce an invariant feature space. This will influence RF predictions and, thus, threshold 8 can be considered as an extreme case.

**Table 5:** The average number of ON bits in b-QAFFPs calculated for HET set compounds

	no AD				AD			
Cutoff	5	6	7	8	5	6	7	8
Average [%]	92.5	53.5	14.5	1.6	71.1	39.4	10.4	1.2
Average [count]	407	235	64	7	313	174	46	5

Model AD was estimated by an ICP with the confidence level of 90% and the maximum interval width, that distinguishes whether the prediction is reliable enough, was set to ± 2.0. Affinities predicted to lie outside model AD were encoded by zeros. b-QAFFP is 440 bits long

QAFFP scaffold hopping potential was assessed for rv-QAFFP constructed from raw data without considering model AD and for b-QAFFP using affinity threshold of 5 and estimating model AD by an ICP substituting missing values (unreliable predictions with interval wider than ± 2.0 at the confidence level of 90%) by zeros. These settings, though suboptimal, are consistent with settings for similarity searching (Tables 1 and 2) and biological activity classification (Table 3).

Out of 1749 existing ACSKs from 22 CLASS data sets, the Morgan2 fingerprint revealed 864 (49%) and the rv-QAFFP fingerprint 911 ACSKs (52%) (Figs. 3, 4, Additional file 4). The differences between Morgan2 and rv-QAFFP (Table 6) are not statistically significant (p-value of two sided Wilcoxon signed-rank paired test is 0.11). On the other hand, the b-QAFFP fingerprint, that unveiled 1065 (61%) ACSKs, performed significantly better (p-value = 1.43e−04 for alternative b-QAFFP > Morgan2 using Wilcoxon signed-rank paired test) than the Morgan2 fingerprint. The highest number of ACSKs (1146. i.e. 66%) was identified when ACSKs found by the rv-QAFFP and b-QAFFP were joined together (further denoted as rv+b-QAFFP). This combination works statistically significantly better than the b-QAFFP fingerprint alone (p-value = 1.43e−04 for alternative b-QAFFP > rv+b-QAFFP using Wilcoxon signed-rank paired test).

**Table 6:** The average number of ACSKs per an assay revealed by the Morgan2, rv-QAFFP and b-QAFFP fingerprints in 22 CLASS sets

	Morgan2	rv-QAFFP	b-QAFFP	rv+b-QAFFP
Average # of ACSKs	39.27 ± 8.25	41.41 ± 8.51	48.41 ± 10.37	52.10 ± 11.12

In addition, the union of ACSKs revealed by both rv-QAFFP and b-QAFFP is reported. Averages are shown together with their standard errors of the mean. Additional file 4 contains detailed information about the number of revealed ACSKs for individual assays

Thus, it may be concluded that while the Morgan2 and rv-QAFFP fingerprints exhibit similarly low scaffold hopping potential, the b-QAFFP fingerprint is better by ca 10%. The highest number of ACSKs was revealed when ACSKs from both rv-QAFFP and b-QAFFP fingerprints were joined together; this combination yielded 17% more ACSKs than the Morgan2 fingerprint.

## Conclusions

We have developed a QSAR-based workflow for the construction of QSAR affinity fingerprint QAFFP. QAFFP is available in two versions: rv-QAFFP (rv- stands for real-valued) and b-QFFP (b- stands for binary). The rv-QAFFP fingerprint consists of biological activities predicted across 440 high-quality assays selected from the ChEMBL19 database and the b-QAFFP fingerprint was constructed by the binarization of the rv-QAFFP fingerprint. The following settings are recommended for the construction of the rv-QAFFP and b-QAFFP fingerprints:

- rv-QAFFP—use predicted bioactivities (i.e., it is not necessary to Z-standardize them) without considering model AD.
- b-QAFFP—to binarize rv-QAFFP values, use the affinity threshold of 5 on the –log scale, estimate model AD by an ICP, substitute missing values (unreliable predictions with interval wider than ± 2.0 at the confidence level of 90%) by zeros.

We would like to stress, that though there exist many tunable settings in QAFFP construction pipeline, our aim was not to optimize each of them for every conceivable application. That would lead to lots of different settings for different use cases which would be rather confusing for the end user. Instead, we decided to propose such QAFFP setting that is robust enough and yields constantly reasonable results. We believe that our published recommendations for the construction of rv- and b-QAFFP fingerprints meet these requirements.

The performance of both QAFFP fingerprints was assessed in three cheminformatics tasks: similarity searching, bioactivity classification and scaffold hopping. In all tasks, the QAFFP fingerprints were compared to 1024 bits long Morgan2 fingerprint (Morgan fingerprint with the radius of 2, an equivalent to the ECFP4 fingerprint) using non-parametric Wilcoxon paired signed-rank test. It was found that the performance of both rv-QAFFP and b-QAFFP fingerprints is similar to that of the Morgan2 (ECFP4) fingerprint in similarity searching and bioactivity classification. However, compared to the Morgan2 fingerprint, the QAFFP fingerprints were able to retrieve significantly higher number of new scaffolds. These findings are rather encouraging given that (i) the QAFFP fingerprints are much shorter, (ii) the QAFFP fingerprints are defined on a purely data-driven fashion, without selecting the targets following biological reasons, and (iii) the models from which the QAFFP fingerprints are derived are far from perfect as their quality is influenced by, for example, QSAR modeling errors [106, 107], experimental errors in publicly available data [108–110], data curation errors [68, 111] or data imputation noise. On the other hand, QAFFP fingerprint is de facto a set of transformed Morgan fingerprints and it, thus, implicitly considers the structure of compounds. For this reason, two structurally similar compounds will show a similar predicted QAFFP profile and possible “activity cliffs” [112, 113] will not be identified.

To conclude, despite the fact that the QAFFP fingerprints are defined on a purely data-driven fashion and that underlying QSAR models rely solely on public data, we have demonstrated that large-scale QSAR modeling [114] is a promising method for the construction of affinity fingerprints. Though affinity fingerprints are inherently noisy, a signal-to-noise ratio is high enough to enable the discovery of bioactive molecules based on biological similarity rather than chemical similarity. In the future, we plan to optimize the composition of the QAFFP fingerprint [115] and to use more biology-informed criteria (e.g., bioactivity data on cancer-related targets are likely to provide high predictive power to find hits eliciting anticancer activity). Future studies will also be needed to investigate the utility of both binary and real-valued QAFFP fingerprints for ligand and target clustering or to evaluate the utility of the QAFFP fingerprint for common computational drug design tasks, including diversity selection, hit expansion, target identification, drug repurposing, and the prediction of adverse side effect.

## Supplementary information

**Additional file 1.** QAFFP construction. Statistics (ligand count and quality criteria $q^{2}$ and $R_{0}^{'2}$) of all 1360 trained models. Each model is given by the target ChEMBL ID and by an activity type (Ki, Kd, IC50 or EC50). 440 models used to construct the QAFFP fingerprint highlighted in green. Ligand data of 440 models (given as ligand ChEMBL ID, SMILES, activity type and activity value).

**Additional file 2.** Similarity searching task. The list of HET and HOM data sets including the number of actives and inactives.

**Additional file 3.** Biological activity classification task. The list of CLASS data sets including the number of actives and inactives.

**Additional file 4.** Scaffold hopping task. The list of targets used for scaffold hopping potential assessment, the number of ACSKs and RACSKs and the number of ACSKs retrieved using the Morgan2, b-QAFFP and rv-QAFFP fingerprints and the combination rv+b-QAFFP.

**Additional file 5.** Similarity searching task. Average values of AUC and EF5 for individual sets in the HET and HOM data sets obtained using various settings (affinity cutoffs of 5-8, AD estimation turned on and off) for the construction of the b-QAFFP fingerprint.

**Additional file 6.** Similarity searching and biological activity classification tasks. Plots showing the performance of the QAFFP and Morgan2 fingerprints.

**Additional file 7.** Biological activity classification task. Average values of AUC and EF5 for individual sets in the CLASS data sets obtained using various settings (affinity cutoffs of 5-8, AD estimation turned on and off) for the construction of the b-QAFFP and rv-QAFFP fingerprints.

## Data Availability

The datasets supporting the conclusions of this article are included as additional files. The source code with the workflow for the generation of QAFFPs is available from GitHub repository: https://github.com/skutac/QAFFPs.

## Abbreviations

ACSK: Active cyclic skeleton
AD: Applicability domain
AUC: Area under the ROC curve
BAF: Bayes affinity fingerprint
BM scaffold: Bemis and Murcko scaffold
b-QAFFP: Binary
QAFFP CCP: Cross-conformal prediction
CLASS: Biological activity classification data sets
CP: Conformal prediction
CSK: Cyclic skeleton
DUD: Directory of useful decoys
ECFP: Extended connectivity fingerprint
EF: Enrichment factor
FN: False negatives
FP: False positives
HET: Heterogeneous data sets
HOM: Homogeneous data sets
HTS: High throughput screening
HTSFP: HTS fingerprint
ICP: Inductive conformal prediction
Morgan2: Morgan fingerprint with the radius of 2
MUV: Maximum Unbiased Validation data sets
QAFFP: QSAR affinity fingerprint
QSAR: Quantitative structure-activity relationship
RACSK: Rich active cyclic skeleton
ROC: Receiver operating characteristic
Sen: Sensitivity
Spe: Specificity
TN: True negatives
TP: True positives
VS: Virtual screening
