# Supplementary material for: QSAR-derived affinity fingerprints (part 1): fingerprint construction and modeling performance for similarity searching, bioactivity classification and scaffold hopping
Source: J Cheminform. 2020 May 29;12:39. doi: 10.1186/s13321-020-00443-6 (PMC7260783; doi:10.1186/s13321-020-00443-6)
Supplement: Supplementary file 6 — Additional file 6. Similarity searching and biological activity classification tasks. Plots showing the performance of the QAFFP and Morgan2 fingerprints. [file 13321_2020_443_MOESM6_ESM.docx]

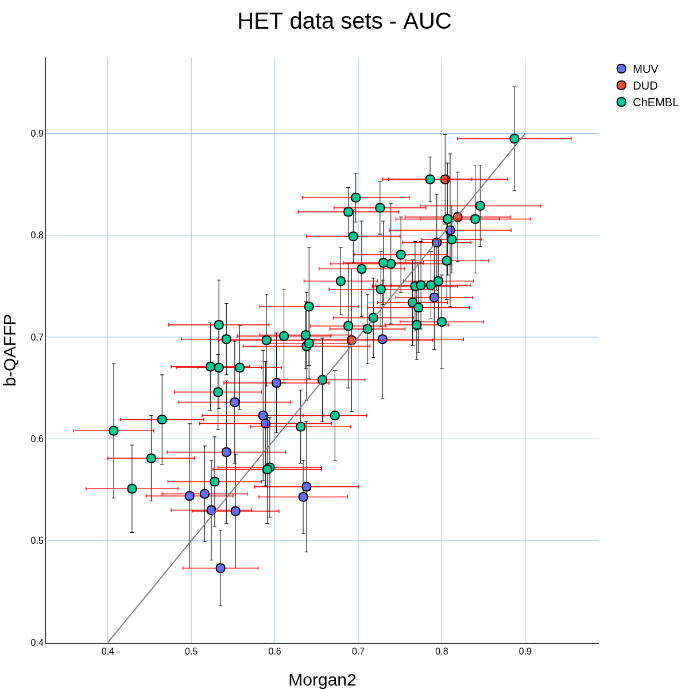

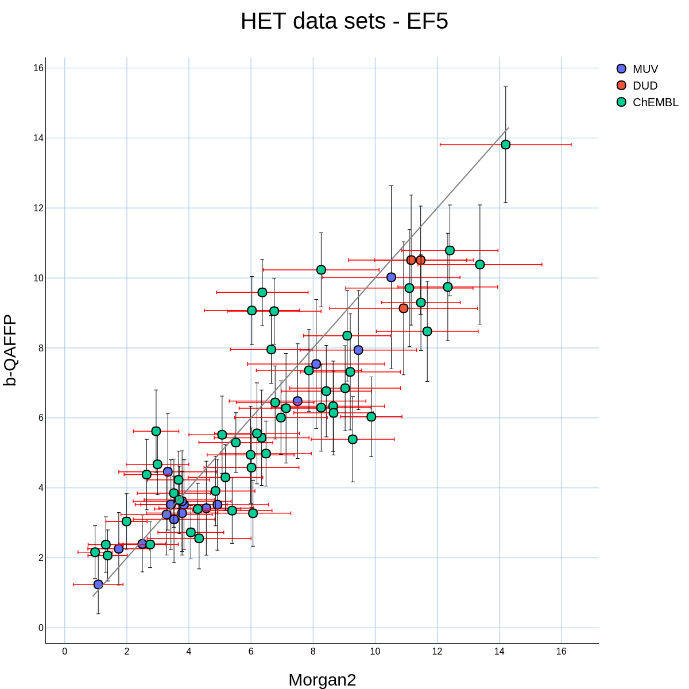


**Figure 1** The performance, given as AUC and EF5, of the Morgan2 (ECFP4) and b-QAFFP fingerprints for similarity searching for 69 HET data sets. The b-QAFFP fingeprint was constructed using affinity cutoff of 5 and taking AD into the account. Error bars depict the standard deviations of predictions calculated over all compounds present in the given data set.


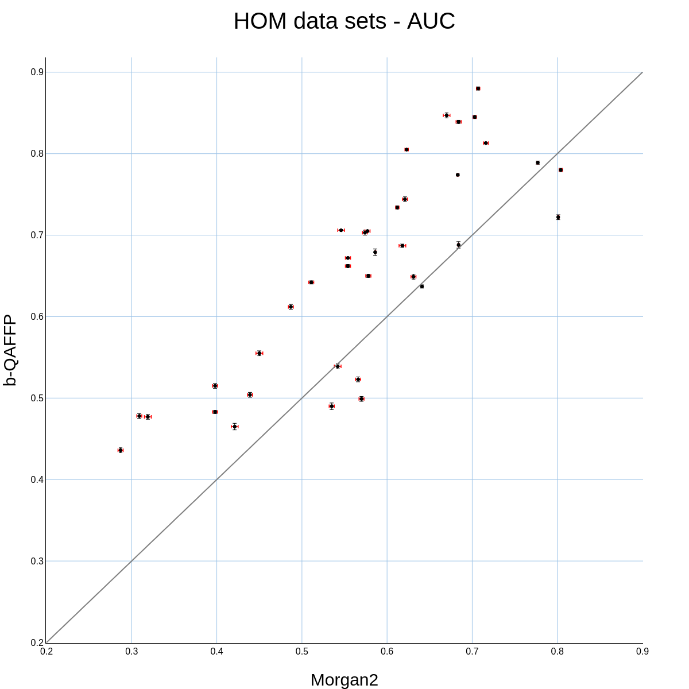

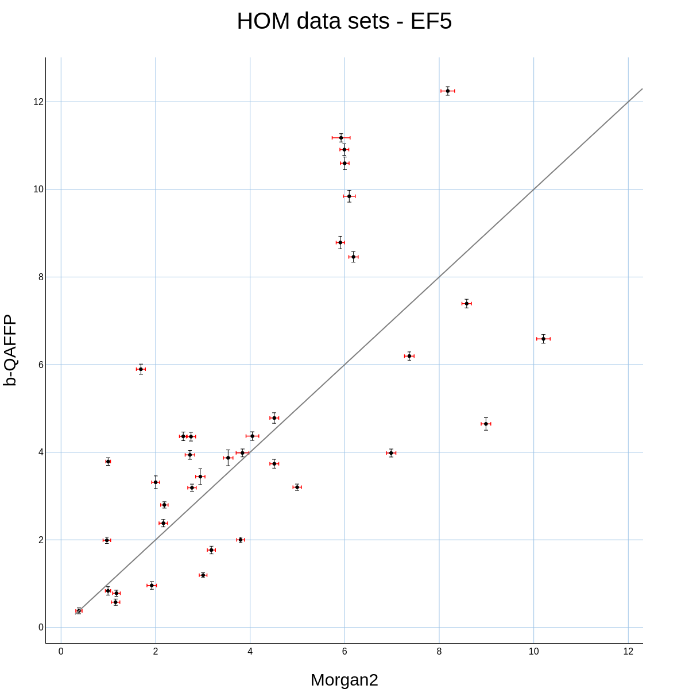


**Figure 2** The performance, given as AUC and EF5, of the Morgan2 (ECFP4) and b-QAFFP fingerprints for similarity searching for 37 HOM data sets. The b-QAFFP fingeprint was constructed using affinity cutoff of 5 and taking AD into the account. Error bars depict the standard deviations of predictions calculated over all compounds present in the given data set.


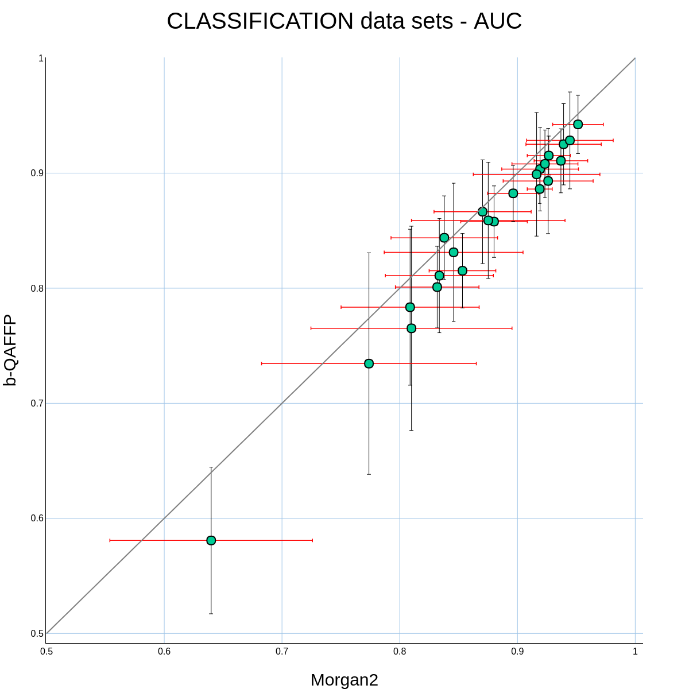

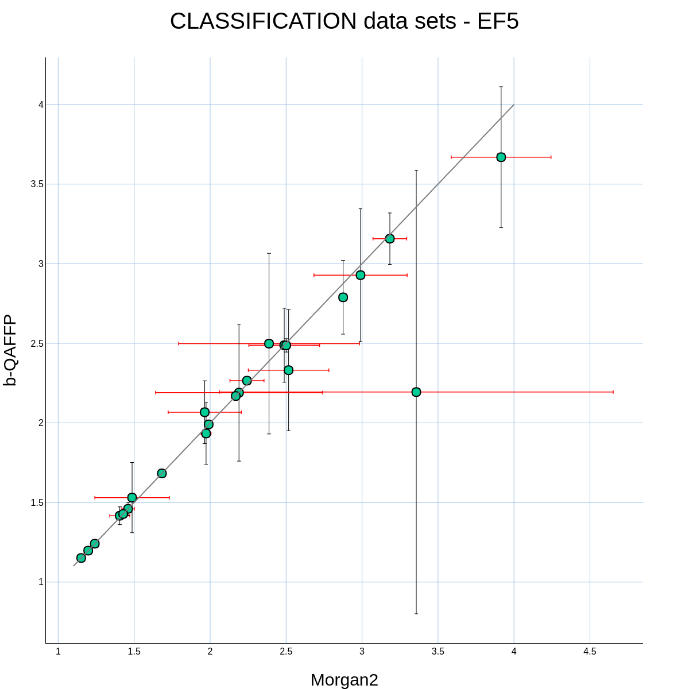


**Figure 3** The performance, given as AUC and EF5, of the Morgan2 (ECFP4) and b-QAFFP fingerprints for biological activity classification for 23 CLASS data sets. The b-QAFFP fingeprint was constructed using affinity cutoff of 5 and taking AD into the account. Error bars depict the standard deviations of predictions calculated over all compounds present in the given data set.


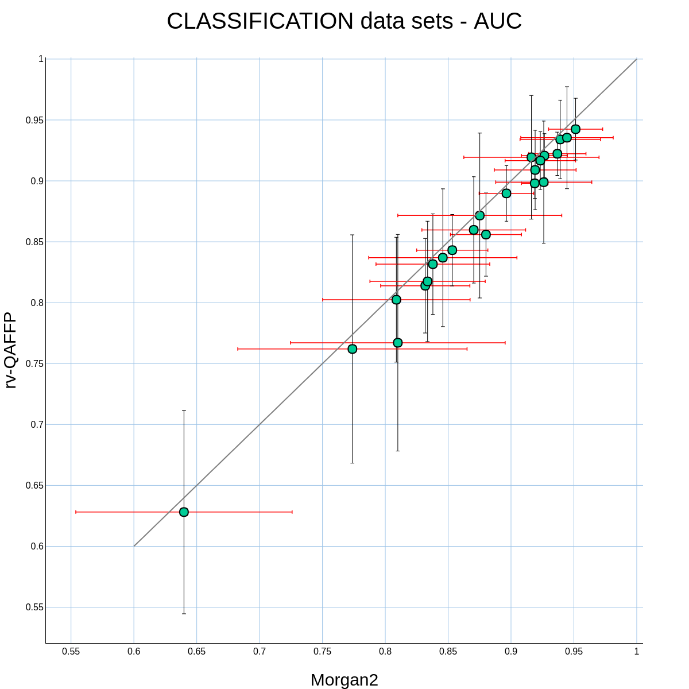

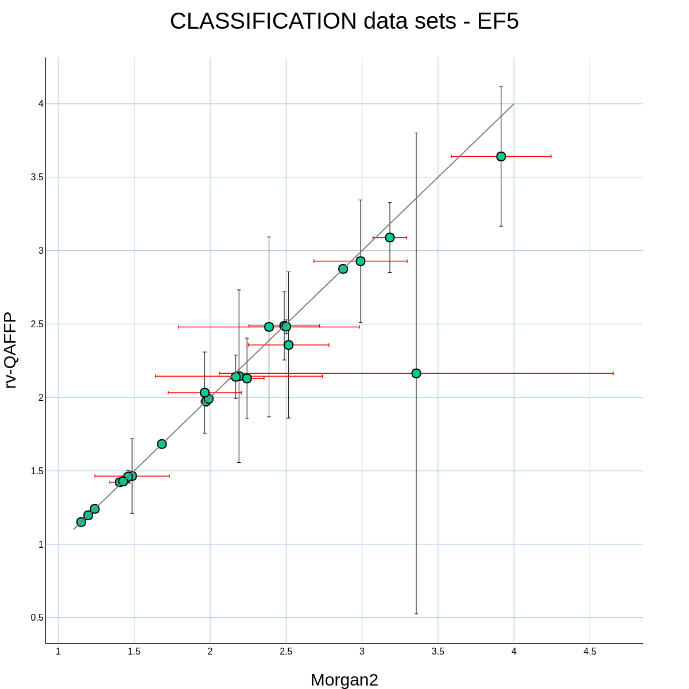


**Figure 4** The performance, given as AUC and EF5, of the Morgan2 (ECFP4) and rv-QAFFP fingerprints for biological activity classification for 23 CLASS data sets. The rv-QAFFP fingeprint was constructed not taking AD into the account. Error bars depict the standard deviations of predictions calculated over all compounds present in the given data set.
